# Scoping review: the current landscape of NIPT in South Africa

**DOI:** 10.1007/s12687-025-00802-6

**Published:** 2025-05-28

**Authors:** Rita Labuschagne, Colleen Aldous, Elana Vorster, Sarah Walters

**Affiliations:** 1 School of Clinical Medicine, University of Kwa-Zulu Natal, Durban, South Africa; 2 Ampath Laboratories, Centurion, South Africa

**Keywords:** Non-invasive prenatal testing (NIPT), South Africa, Prenatal screening, Low-middle income countries (LMIC), Genetic counselling

## Abstract

Non-invasive prenatal testing (NIPT) analyses cell-free fetal DNA (cffDNA) in maternal blood to screen for trisomies 13, 18, and 21, offering critical insights into common chromosomal aneuploidies without requiring invasive procedures. Advances in technology have made NIPT faster, more affordable, and widely accessible in many high-income countries (HICs), where it has been integrated into routine prenatal care. However, in low- and middle-income countries (LMICs), including South Africa (SA), NIPT remains largely inaccessible. It is available only to individuals who can afford out-of-pocket expenses or those with medical aid and high-risk pregnancies, limiting its potential impact on broader public health. This scoping review aimed to examine the current landscape of NIPT in SA and other LMICs, with a focus on identifying key themes, implementation challenges, and gaps in the literature. Thematic analysis was used to synthesise findings across studies. The review followed PRISMA guidelines, where relevant literature was identified through database searches using the Boolean term: (NIPT OR "Non-invasive prenatal screen*" OR "Non-invasive prenatal test*" OR NIPD) AND (South Africa* OR "low-middle income*" OR "LMIC"). Inclusion and exclusion criteria were applied to ensure relevance and quality. Twenty-nine articles were included in the review, and the following key themes were identified: (1) NIPT is a screening, not a diagnostic, test; (2) pre- and post-test genetic counselling is recommended; (3) NIPT is expensive and complex; (4) implementation is challenging; (5) ethical, legal, and social concerns exist; (6) access remains unequal; (7) NIPT reduces the need for invasive testing due to high sensitivity and specificity; (8) NIPT should complement, not replace, first-trimester screening; (9) screening for other genetic conditions may be more relevant in LMICs; and (10) NIPT benefits HIV-positive mothers. Widespread NIPT implementation in SA is constrained by cost, infrastructure, and competing healthcare priorities. While next-generation sequencing (NGS)-based NIPT remains expensive, alternative technologies, such as droplet-based NIPT, may offer a more affordable approach for high-risk pregnancies, reducing reliance on invasive procedures. NIPT holds significant potential to improve prenatal care in SA. However, its current inaccessibility within the public sector and among underserved populations underscores critical gaps in equity, implementation, and broader prenatal screening practices, which remain inadequate across much of the country. A locally validated, cost-effective alternative such as droplet digital PCR-based NIPT (ddNIPT) could help bridge this divide and make advanced screening more accessible and sustainable. Future research must prioritise validating such alternatives within the South African context. Ensuring equitable access to NIPT is essential to improving outcomes for all pregnant women.

**Supplementary Information:**

The online version contains supplementary material available at 10.1007/s12687-025-00802-6.

## Introduction

Non-invasive prenatal testing (NIPT) refers to a molecular screening test that can be performed during pregnancy at no risk to the fetus. During pregnancy, maternal blood contains cell-free fetal DNA (cffDNA), which can be tested for specific chromosomal abnormalities in the fetus, using advanced NIPT Next Generation sequencing (NGS) technology. NIPT has been commercially available for over a decade (Chetty et al. 2013; Dan et al. 2012) and serves as a supplementary prenatal screening tool alongside biochemical maternal serum screening. NIPT screens for trisomy 13, 18, and 21, as well as sex chromosome aneuploidies and fetal sex, although whole genome screening is also now available. NIPT analyses fetal and maternal cell-free DNA (cfDNA) from a maternal blood sample, where an abnormal chromosome fragment ratio suggests fetal aneuploidy (Benn & Cuckle 2023). Biochemical maternal serum screening measures placental and fetal proteins in maternal blood, combined with ultrasound findings and maternal age, to assess the risk of fetal abnormalities, including trisomies and neural tube defects (NTDs) (Chasen 2014).

NIPT has a higher sensitivity and specificity for the fetal abnormalities trisomy 13, 18, and 21 than conventional biochemical maternal serum screening. Biochemical maternal serum screening has an estimated sensitivity and specificity for trisomy 21 of 78.9% and 94.6%, respectively. The sensitivity and specificity of NIPT for trisomy 21 are 99.6% and 100%, respectively (Badeau et al. 2017). This superior performance makes NIPT highly advantageous for prenatal screening, as it substantially reduces the rate of false-positive results and, consequently, the number of unnecessary invasive follow-up procedures such as chorionic villus sampling (CVS) or amniocentesis. While all screening tests with positive results require diagnostic confirmation, the high negative predictive value of NIPT minimises the need for confirmatory invasive testing in low-risk cases, thereby reducing procedure-related risks for both the pregnant mother and the fetus. Compared with conventional biochemical maternal serum screening, NIPT is more costly, has a slightly longer turnaround time, and is typically performed in centralised laboratories using intricate and expensive NGS technology. In contrast, biochemical maternal serum screening is widely accessible, integrated into routine prenatal care, and generally covered by medical insurance providers.

South Africa (SA) works on a dual healthcare system where more than 84% of the population relies on state funding, i.e. facilities and tests provided by the state, or public healthcare (Department of Health 2017; Statistics South Africa 2023a). The remaining 15.7% of the population obtains private healthcare where members contribute towards a medical fund, or medical aid, which enables private medical care subject to the medical aid rules (Statistics South Africa 2023a).

Many high-income countries (HICs) have adopted NIPT in their prenatal screening workup, with government funding or patient reimbursement (Griffin et al. 2018; Hui & Hyett 2013; Jayashankar et al. 2023; Minear et al. 2015a, b; Minear et al. 2015a, b; Ravitsky et al. 2021). In contrast, NIPT remains largely inaccessible in low- and middle-income countries (LMICs), including SA, where it is not covered by government funding and where there is limited funding from medical aids for private patients (Allyse et al. 2015; Jayashankar et al. 2023; Minear et al. 2015a, b; Mozersky et al. 2017; Noh et al. 2019; Urban et al. 2014). Some private medical aids cover high-risk pregnancies or it needs to be paid out of pocket (Jayashankar et al. 2023; Ravitsky et al. 2021). High-risk pregnancies include pregnancies of advanced maternal age (AMA), pre-existing medical conditions, high-risk biochemical maternal serum screen, the detection of sonar abnormalities, etc.

The American College of Obstetricians and Gynecologists (ACOG) stated that NIPT has a higher sensitivity and specificity than other screening tests and that all pregnant women should be offered prenatal screening (American College of Obstetricians and Gynecologists 2017). With the rapid advancement of NIPT technology, many HICs are integrating NIPT into routine prenatal care. Understanding SA’s position regarding NIPT availability and techniques is crucial for assessing its readiness to adopt these advances and to improve prenatal screening practices in SA. Therefore, we conducted a scoping review to assess the current landscape, status and challenges of implementing NIPT in SA. This will help determine whether current prenatal screening programmes can be adapted to improve NIPT accessibility for all pregnant women.

## Aim

This scoping review aimed to explore the current landscape of NIPT in SA and other LMICs. Specifically, it sought to identify key themes, challenges, and gaps in the literature regarding the implementation, accessibility, and availability of NIPT—with a particular focus on the South African context. The findings are intended to inform future research and guide the development of equitable and effective prenatal screening strategies in SA.

## Methods

The central research question guiding this scoping review was: What is the current landscape of NIPT and non-invasive prenatal screening availability in South Africa, and what are the existing gaps and challenges in its implementation and accessibility?

The scoping review was conducted using the PRISMA guidelines (Page et al. 2021). The following five databases were identified and searched: Pubmed, Medline, BioMed Central, EBSCO Resource EBSCOhost Web and CINAHL Resource EBSCO Discovery Service. There was no beginning date limit to the search. Articles published until 30th June 2024 were included. Additional articles were searched for via Google and Google Scholar to ensure no relevant articles were missed.

Databases were searched with the following Boolean term: (NIPT OR"Non-invasive prenatal screen*"OR"Noninvasive prenatal screen*"OR"Noninvasive prenatal test*"OR"Non-invasive prenatal test*"OR NIPD) AND (South Africa* OR'low-middle income* OR"LMIC"). Articles were downloaded and sorted, making use of Zotero referencing software. The inclusion and exclusion criteria are summarised below.

Inclusion criteria:

- The article discusses NIPT as a prenatal screening tool.
- The article is available in the English language.
- Full-text article is available.
- The article was published in a peer-reviewed journal.

Exclusion criteria:

- The article only discusses RHD genotyping.
- The article only discusses using NIPT for single-gene disorders.
- The article is not accessible.
- The article is not published.
- The article is not written in the English language.
- The article is part of grey literature, i.e. thesis, magazine articles, clinical trials, books, or documents.

The primary author (RL) conducted an initial screening of article titles to assess relevance. Articles that did not reference NIPT, cfDNA, SA, LMICs or prenatal screening were excluded. In the second round of screening, all authors participated in reviewing article titles and abstracts to determine relevance. In cases where consensus could not be reached, articles were retained for further consideration. The final screening was conducted by RL, who read and analysed all remaining articles to identify recurring themes and patterns. A thematic analysis approach guided the process of identifying themes and key insights across the literature (Braun and Clarke 2006). Articles were examined multiple times to ensure a comprehensive understanding of the content. Key findings, trends, and notable issues were systematically documented. Comparative analysis was conducted to identify similarities and differences across the literature, and recurring concepts were grouped to develop preliminary themes. These themes were subsequently reviewed and refined to ensure consistency, relevance, and comprehensive coverage of all topics.

## Results

**Fig. 1:**
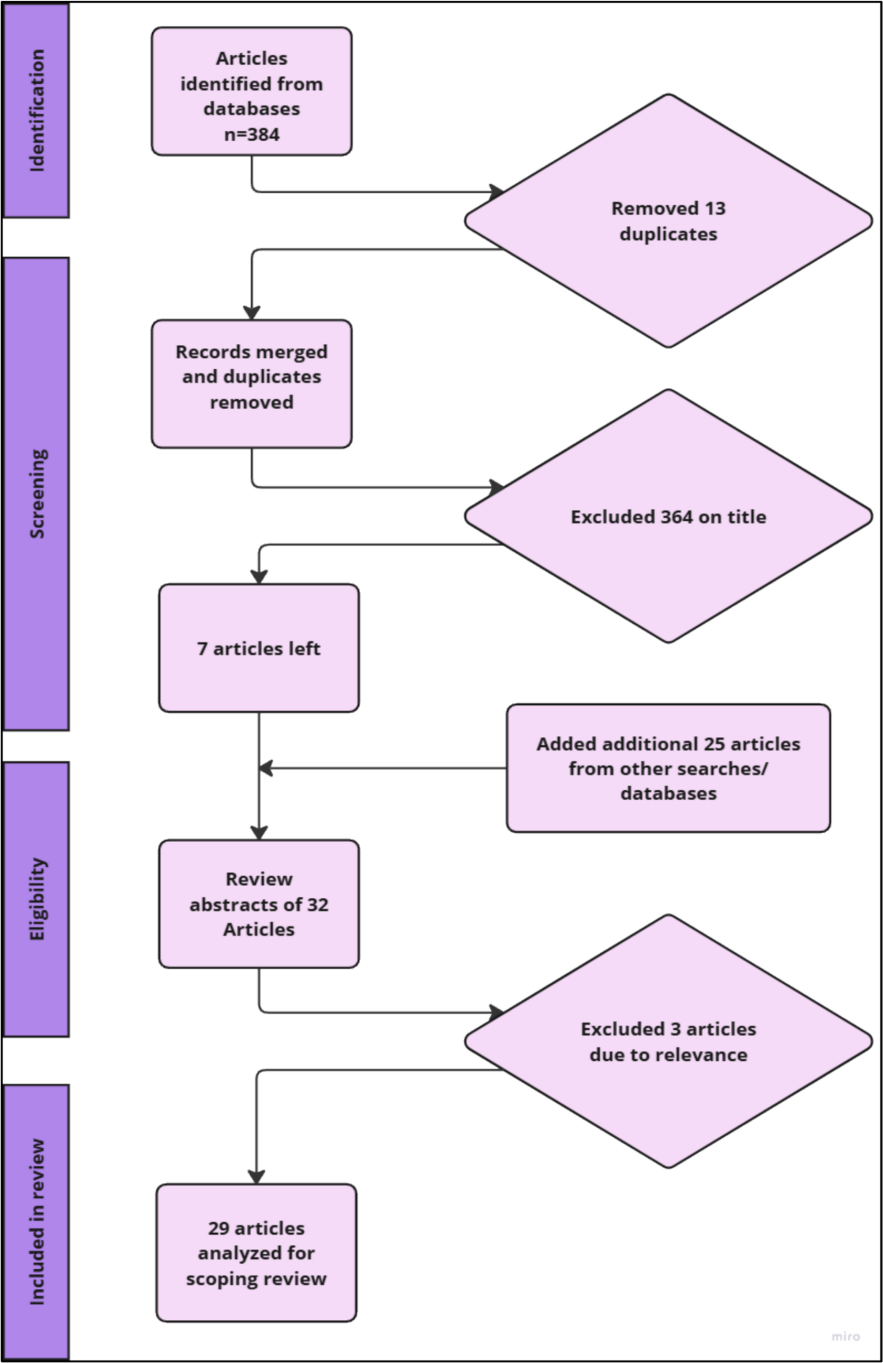
Prisma flow diagram summarising literature selection process

The initial search across the specified databases identified 384 articles. After removing 13 duplicates, 371 articles remained. The author then screened the titles, excluding 364 articles that did not meet the criteria. An additional 25 articles were identified through supplementary searches, using Google and Google Scholar, resulting in a total of 32 articles for further evaluation. These 32 articles were reviewed by all authors based on their titles and abstracts, leading to the exclusion of 3 articles due to lack of relevance. Ultimately, 29 articles were included in this scoping review. The process is described in Fig. 1.

### Timeline of articles

**Fig. 2:**
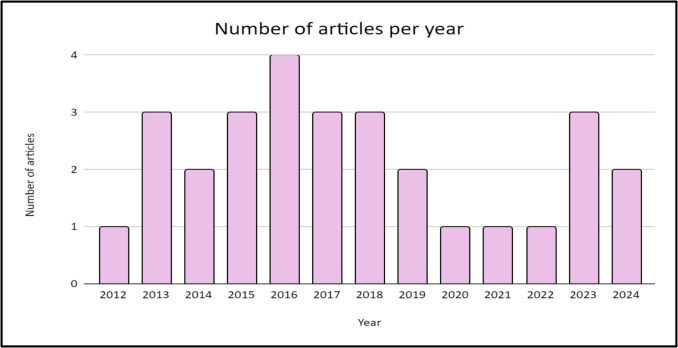
Chart showing the period when the 29 relevant articles were published

The first article discussing NIPT was published in 2012, and most articles (*n* = 4) were published in 2016, with the number of relevant articles gradually decreasing. Figure 2 shows the period during which the 29 articles were published. This emphasises the lack of literature on NIPT in SA.

Of the 29 articles obtained, 11 specifically mentioned SA (Bhorat et al. 2018; Jayashankar et al. 2023; Minear et al. 2015a, b; Mnyani et al. 2016; Mossfield et al. 2022; Mozersky et al. 2017; Sium et al. 2023; Soster et al. 2024, p. 20; Thaldar 2023; Urban et al. 2014; Ventura et al. 2013). The remaining 18 articles in this scoping review discussed the implementation of NIPT, specifically in LMICs, its benefits, challenges, limitations and ethical issues.

### Themes

The scoping review identified 10 themes through analysing the 29 articles. The themes have been grouped and are summarised in Table 1, indicating how many articles discussed and addressed the themes.

**Table 1:** Themes identified

Theme addressed	Number of articles
**NIPT is a screening test**	17
*The need for invasive testing will decrease as NIPT offers higher sensitivity and specificity than conventional screening*	9
*NIPT cannot/should not replace combined first-trimester screening*	4
*NIPT is advantageous for HIV-positive mothers*	1
**The process of implementing NIPT is multifaceted and challenging**	21
*NIPT testing is expensive and complex*	20
*Screening for other genetic disorders might be more relevant in LMICs than trisomies 13, 18 and 21*	2
**Ethical, Legal, and Social Issues**	15
*Pre- and post-test counselling and informed consent are essential for NIPT*	18
*Unequal accessibility of NIPT*	11

#### NIPT is a screening test

NIPT remains a screening test, and although it is more sensitive and specific than biochemical maternal serum screening, invasive testing is still recommended to confirm the NIPT results to obtain a diagnostic result (Allyse et al. 2015; Badeau et al. 2017; Bhorat et al. 2018; Brady et al. 2016; Griffin et al. 2018; Haidar et al. 2018; Hui & Hyett 2013; Jayashankar et al. 2023; Minear et al. 2015a, b; Mnyani et al. 2016; Mossfield et al. 2022; Mozersky et al. 2017; Noh et al. 2019; Nshimyumukiza et al. 2018; Sánchez-Durán et al. 2019; Swanson et al. 2013; Urban et al. 2014). Since NIPT analyses cfDNA, a mixture of maternal and fetal DNA, results may be affected by maternal conditions, low fetal fraction, or placental mosaicism, leading to false positive or negative results (Griffin et al. 2018). In addition, the fetal DNA originates from the placenta rather than directly from the fetus, therefore NIPT remains classified as a screening rather than a diagnostic test. The number of false positive or negative results is below 0.4% and 0.004% respectively for trisomy 21 (Badeau et al. 2017), and are caused mainly by confined placental mosaicism. (Brady et al. 2016; Griffin et al. 2018; Mossfield et al. 2022; Soster et al. 2024; Swanson et al. 2013).

#### The need for invasive testing will decrease

Nine articles highlight that the need for invasive testing will decrease as NIPT offers higher sensitivity and specificity (Allyse et al. 2015; Brady et al. 2016; Griffin et al. 2018; Jayashankar et al. 2023; Kelly & Farrimond 2012; Minear et al. 2015a, b; Noh et al. 2019; Ravitsky et al. 2021; Swanson et al. 2013). Some studies have reported a decline in invasive testing, which is generally beneficial for maternal and fetal health. Invasive procedures carry risks such as miscarriage, infection, and psychological stress, even though complication rates are low. Non-invasive procedures will reduce maternal anxiety and risk to the fetus as well. However, some argue that the decreased frequency of such procedures could reduce practitioners’ expertise, potentially increasing miscarriage risks due to procedural complications.

#### NIPT cannot/should not replace combined first-trimester screening

Although NIPT has a higher sensitivity and specificity for trisomy 13, 18, and 21 than first-trimester biochemical maternal serum screening, it should not replace it (Allyse et al. 2015; Badeau et al. 2017; Hui & Hyett 2013; Minear et al. 2015a, b). NIPT is limited to only screening for specific genetic abnormalities. Biochemical screening (such as PaPP-A) can also indicate risk for pre-eclampsia, neural tube defects, intrauterine growth restriction, and fetal demise. (Allyse et al. 2015; Hui & Hyett 2013; Tan 2015; Tran 2006).

Combination screening, consisting of biochemical maternal serum screening, ultrasound and NIPT is the best approach for prenatal screening since only 44–64% of fetal chromosome abnormalities include trisomy 13, 18 and 21, which are detectable by NIPT (Badeau et al. 2017).

#### NIPT is advantageous for HIV-positive mothers

One South African article highlights that NIPT testing will protect the fetus if the mother is HIV positive, since invasive CVS or amniocentesis will put the fetus at risk of obtaining HIV (Urban et al. 2014). This is advantageous in populations with a high HIV prevalence.

#### The process of implementing NIPT is multifaceted and challenging

The implementation of NIPT is discussed in twenty-one articles since there are no universal guidelines for how, where and when to implement and request testing (Allyse et al. 2015; Bhorat et al. 2018; Carbone et al. 2020; Gitsels - Van Der Wal et al. 2014; Griffin et al. 2018; Haidar et al. 2018; Hui & Hyett 2013; Jayashankar et al. 2023; Kelly & Farrimond 2012; Minear et al. 2015a, b; Minear et al. 2015a, b; Mozersky et al. 2017; Noh et al. 2019; Nshimyumukiza et al. 2018; Ravitsky et al. 2021; Sánchez-Durán et al. 2019; Sium et al. 2023; Soster et al. 2024, p. 20; Swanson et al. 2013; Urban et al. 2014; Ventura et al. 2013).

“The process of integrating this test into the practical clinical environment needs more studies and should be determined with the prudent assessment.”(Noh et al. 2019).

Two articles (Allyse et al. 2015; Hui & Hyett 2013) explore the most cost-effective approach to implementing NIPT, although numerous factors must be considered. These factors include the capacity of the healthcare system, the presence of a prenatal screening programme, the availability of public funding, and the broader social, cultural, legal, and political landscapes (Ravitsky et al. 2021).

#### NIPT testing is expensive and complex

NIPT testing is expensive compared with other prenatal screening tests. Twenty articles discuss how expensive NIPT is and that it is not accessible to many in the public sector when self-payment is required (Allyse et al. 2015; Badeau et al. 2017; Bhorat et al. 2018; Brady et al. 2016; Carbone et al. 2020; El Khattabi et al. 2016; Griffin et al. 2018; Haidar et al. 2018; Hui & Hyett 2013; Jayashankar et al. 2023; Minear et al. 2015a, b; Minear et al. 2015a, b; Mozersky et al. 2017; Noh et al. 2019; Nshimyumukiza et al. 2018; Ravitsky et al. 2021; Sánchez-Durán et al. 2019; Sium et al. 2023; Urban et al. 2014; Ventura et al. 2013). NIPT’s high cost makes it impractical to include in the public healthcare prenatal screening system, especially in LMICs. Reducing NIPT prices over time would make NIPT and pregnancy screening more accessible and feasible.

“Although NIPT is more preferable than genetic amniocentesis or CVS for screening these serious conditions, the technical know-how about the testing and unavailability of skilled personnel make it inaccessible in low-income countries, compared to the invasive ones”(Sium et al. 2023). NIPT relies on NGS technology, which is costly and technically complex. It generates large volumes of data that require analysis and interpretation using specialised software and trained scientists. As a result, NIPT is typically performed in large, centralised laboratories, limiting its accessibility.

#### Screening for other genetic disorders might be more relevant in LMICs compared with trisomy 13, 18 and 21

Single gene disorders such as hemoglobinopathies, including sickle cell anaemia and beta-thalassemia, are prevalent in Western Africa and its neighbouring countries. Focusing on disease prevalence in LMICs might be more beneficial for these countries (Allyse et al. 2015; Mozersky et al. 2017).

### Ethical, legal, and social issues

Informed choice, sex selection, termination of pregnancy, economic inequality, eugenics and possible discrimination against the disabled are some of the issues that have been identified concerning NIPT (Allyse et al. 2015; Gitsels - Van Der Wal et al. 2014; Griffin et al. 2018; Haidar et al. 2018; Hui & Hyett 2013; Jayashankar et al. 2023; Kelly & Farrimond 2012; Ladak et al. 2024; Minear et al. 2015a, b; Minear et al. 2015a, b; Mozersky et al. 2017; Ravitsky et al. 2021; Sium et al. 2023; Thaldar 2023; Ventura et al. 2013). Incidental findings in the mother, such as maternal malignancy or sex chromosome aneuploidy findings in the fetus, which do not always have a visible phenotype, could complicate counselling and are additional ethical challenges accompanying NIPT (Minear et al. 2015a, b).

#### Pre- and post-test counselling and informed consent are essential for NIPT

Eighteen articles emphasised the critical role of pre- and post-test counselling in NIPT (Allyse et al. 2015; Badeau et al. 2017; Bhorat et al. 2018; Griffin et al. 2018; Haidar et al. 2018; Hui & Hyett 2013; Jayashankar et al. 2023; Kelly & Farrimond 2012; Minear et al. 2015a, b; Minear et al. 2015a, b; Mnyani et al. 2016; Mozersky et al. 2017; Noh et al. 2019; Ravitsky et al. 2021; Smith et al. 2018; Swanson et al. 2013; Urban et al. 2014; Ventura et al. 2013). Patients must be adequately informed to make autonomous decisions about undergoing NIPT. Informed consent is essential, as NIPT should not be routinised as a standard prenatal test. Its results may have complex and far-reaching implications, potentially revealing information about the fetus or pregnancy that the patient did not specifically seek. Pre- and post-test counselling provided by a healthcare professional or genetic counsellor should ensure that patients gain a clear understanding of the advantages and limitations of NIPT, enabling them to make well-informed decisions while safeguarding their autonomy.

#### Unequal accessibility of NIPT

NIPT has been implemented in over 90 countries (Minear et al. 2015a, b). However, the implementation and accessibility of this highly effective screening test are unequal worldwide (Allyse et al. 2015; Haidar et al. 2018; Hui & Hyett 2013; Jayashankar et al. 2023; Minear et al. 2015a, b; Minear et al. 2015a, b; Mnyani et al. 2016; Mozersky et al. 2017; Ravitsky et al. 2021; Urban et al. 2014; Ventura et al. 2013). In most HICs, NIPT is publicly funded or reimbursed. In SA and other LMICs, it is largely limited to those paying out of pocket or select high-risk pregnancies covered by medical aid. As a result, access is primarily restricted to wealthier individuals. This could potentially lead to a relatively higher burden of common chromosomal disorders in lower-income populations compared with middle- and high-income groups.

## Discussion

The 29 scoping review articles indicate the current landscape and status of NIPT in SA and other LMICs. Only 11 articles specifically mention SA, which indicates a gap in the literature for NIPT in SA. Of those 11 articles, only three are South African published articles, specifically discussing SA, NIPT, its clinical use, value, and challenges. (Bhorat et al. 2018; Mnyani et al. 2016; Urban et al. 2014). These articles are outdated and have been published between 2014 and 2018. One of the articles also reiterates the need for more NIPT studies applicable to SA and LMIC to assess the value and role of NIPT (Mnyani et al. 2016).

The identified themes are discussed below in relation to existing challenges, opportunities, and priorities within South Africa’s prenatal care framework. The discussion considers the current state of prenatal screening in SA and situates the findings within the broader South African healthcare landscape.

### NIPT is a screening test

#### NIPT's potential role as a screening test in the South African public healthcare sector

In SA, the accessibility to prenatal screening tests is unequal across the public sector. Pregnant women, especially those who reside in rural communities, do not have access to prenatal screening, which includes biochemical screening, ultrasound, and NIPT.

In a South African study, 45% of expectant mothers believed that the only reason they should go to a clinic while pregnant is to get an antenatal card to record essential maternal and fetal health information (Haddad et al. 2016). The most common screening technique in the public sector to determine a high-risk pregnancy is maternal age, since other resources like sonar and biochemical screening are scarce and expensive (Geerts 2008; Urban et al. 2011).

According to the Clinical Guidelines for Genetics Services in SA, published in November 2021, maternal serum screening is only available at district hospitals and not at primary health care level such as in clinics or community health centres. Therefore, an additional screening method, like NIPT, might not be appropriate, justified, or applicable for SA.

However, in a study conducted in SA, the average gestational age at which pregnant women first presented at a public healthcare facility was 19.1 weeks (Haddad et al. 2016), which is considerably later than the recommended 12-week gestation mark for initiating antenatal care (World Health Organization 2016). This delay has been attributed to a variety of factors, including fear of HIV testing, cultural beliefs, and uncertainty regarding the continuation of the pregnancy (Haddad et al. 2016). Other factors contributing to delays may include limited access to clinics, travel-related costs, and the need to take time off work, whether paid or unpaid. Given this delayed presentation, the implementation of biochemical screening would likely be of limited utility in this population, as such screening is typically only possible and accurate up to 20 weeks of gestation (Driscoll & Gross 2009). NIPT does not have a strict gestational age cut-off, which makes it particularly advantageous for women who present later in pregnancy and still require prenatal screening.

Another aspect to consider is that because NIPT is a screening test, positive results should be confirmed with invasive testing. The time frame, gestational age, patient informed consent, follow-up time, availability of resources and results must be considered when choosing the most appropriate prenatal test. For example, if a woman is 18 weeks pregnant and her fetus is determined to be at high risk for aneuploidy, and she would consider termination if the fetus were affected with a common trisomy, invasive testing will give a quicker and definitive answer, compared with NIPT.

The South African Society for Ultrasound in Obstetricians and Gynaecologists (SASUOG) and the South African Society of Obstetricians and Gynaecologists (SASOG) have stated that the majority fetal anomalies in SA are diagnosed after 20 weeks (The South African Society for Ultrasound in Obstetricians and Gynaecologists (SASUOG) & The South African Society of Obstetricians and Gynaecologists (SASOG) 2015).Therefore, it is important to refer to the termination of pregnancy (TOP) act in SA and how it should be applied.

The TOP act in SA specifies that a woman can terminate a pregnancy until 20 weeks of gestational age under specific conditions. The circumstances stipulated include; if the continued pregnancy poses a risk to the woman's physical or mental health, if there is a substantial risk that the fetus would suffer from a severe physical or mental abnormality, if the pregnancy resulted from rape or incest or if continuing the pregnancy would significantly affect the woman's social or economic circumstances. Termination can only occur after 20 weeks under exceptional circumstances, such as if the continued pregnancy would endanger the woman's life, if there is a risk of severe malformation of the fetus, or if there is a risk of injury to the fetus (National Department of Health,RSA 2019). SASUOG, with the endorsement of SASOG, has issued additional guidelines concerning the termination of pregnancy for fetuses affected by anomalies after 20 weeks (The South African Society for Ultrasound in Obstetricians and Gynaecologists (SASUOG) & The South African Society of Obstetricians and Gynaecologists (SASOG) 2015). These guidelines clarify the gestational limits and the severity for which termination is considered appropriate.

### The need for invasive testing will decrease

The Green Top Guideline on Amniocentesis and CVS from the Royal College of Obstetricians and Gynaecologists recommends that medical practitioners perform a minimum of 20 invasive procedures per year to maintain competency (Navaratnam et al. 2022). Although NIPT has significantly reduced the need for invasive testing, positive or high-risk NIPT results should be confirmed through diagnostic procedures such as amniocentesis or CVS. With the adoption of NIPT, which has a low false-positive rate, the demand for invasive testing has declined, particularly in HICs where NIPT has been integrated into routine prenatal care. The reduction in the volume of amniocentesis procedures has been linked to an increase in procedure-related complications, including a higher incidence of miscarriage. Studies have demonstrated an inverse correlation between the number of invasive procedures performed and the risk of fetal loss following amniocentesis, suggesting that decreased practitioner experience may contribute to a higher risk of procedure-related miscarriage (Hui et al. 2015).

In SA, particularly within the public healthcare sector, invasive testing, especially amniocentesis, remains the primary method for prenatal diagnosis, largely due to limited or no availability of NIPT (Allyse et al. 2015; Sium et al. 2023).

Invasive procedures like CVS and amniocentesis require expertise, and few doctors are qualified to perform them. In rural areas, access to skilled practitioners is limited, further restricting availability of invasive testing (Allyse et al. 2015). This highlights the need for improved access to NIPT in the public sector.

NIPT is a screening test and requires invasive diagnostic testing for confirmation of a high-risk result. Therefore, invasive testing remains essential. The following quote emphasises this: “The frequency of chromosome abnormalities, which would be missed if invasive prenatal chromosomal microarray-based analysis would be replaced by targeted NIPT, has been estimated to be 16.9%, including 2% of those pregnancies deemed at high risk of aneuploidy based upon abnormal serum screening results.”(Brady et al. 2016). However, chromosomal microarray analysis is not currently available in the public sector in SA and remains limited within the private sector. These findings reinforce the view that NIPT should not replace biochemical screening, as approximately 2% of high-risk pregnancies may go undetected using NIPT alone.

### NIPT cannot/should not replace combined first-trimester screening

In SA, biochemical screening has several advantages. Testing is not labour intensive, has a quick turnaround time, the test provides additional information about the pregnancy and the sample is collected in a more easily available sample tube that does not have to reach the laboratory within a set time. Despite these benefits and the reduced costs of reagents, equipment, and technology compared to NIPT, this fundamental screening test, which is routinely available in HICs, remains largely inaccessible within the South African public healthcare sector.

### NIPT is advantageous for HIV-positive mothers

HIV remains a significant public health issue in SA, with a prevalence of 13.7%. Almost one in four women of reproductive age (15–49 years) are living with HIV (Statistics South Africa 2022). In this context, NIPT is particularly valuable. Unlike invasive procedures such as CVS or amniocentesis, NIPT does not involve breaching the uterine environment and therefore avoids the risk of transplacental infection and vertical HIV transmission (Hui et al. 2015). NIPT is especially relevant for women who may not yet have achieved full viral suppression or who face barriers to consistent antiretroviral treatment.

Previous studies have shown higher rates of mother-to-child transmission (MTCT) of HIV following invasive procedures such as amniocentesis, particularly when maternal viral load is not well controlled (Mandelbrot et al. 2009). The risk of transmission is closely linked to the viral load, and MTCT becomes very low when the viral load is undetectable (Navaratnam et al. 2022). In SA, the MTCT rate has been reported at around 0.9% (Goga et al. 2018) though this estimate does not account for the potential increase when invasive testing is performed.

Beyond lowering the risk of MTCT, NIPT may also reduce the need for invasive follow-up procedures, support earlier decision-making, and contribute to better prenatal care planning. For HIV-positive pregnant women, particularly in high-prevalence settings like SA, these advantages make NIPT a safer and more accessible option.

### The process of implementing NIPT is multifaceted and challenging

Some of the limitations to implementing NIPT in SA include the viability of the sample, expensive collection tubes with short expiry dates, transport and cost.

The Clinical Guidelines for Genetic Services in South Africa recommend offering NIPT to all women with high-risk pregnancies (National Department of Health: Pretoria, South Africa 2021). This includes cases of AMA, previous pregnancies affected by chromosomal abnormalities, ultrasound-detected abnormalities or pregnancies showing an increased biochemical risk. However, the guidelines also state that NIPT should first be introduced as a pilot study before widespread implementation. While these recommendations indicate that the South African government has considered the integration of NIPT, to the author’s knowledge, no studies have been conducted to date to explore its feasibility or implementation.

Several articles debate when and how NIPT should be implemented, as it is a more expensive test than biochemical screening. Most studies focused on high-risk pregnancies, skewing the results of the positive predictive value of the test. Women with a high-risk biochemical screen have previously been offered NIPT in SA. However, the uptake was low, with only 2.3% of women participating in this study (Mnyani et al. 2016). One possible reason for the low uptake of NIPT is that, in 2016, samples were sent overseas for analysis, resulting in delayed turnaround times. It is also assumed that patients had to pay out of pocket, when potentially few expectant couples could afford this.

A South African article suggests that NIPT screening should be extended to pregnant women in the intermediate-risk category (Bhorat et al. 2018). The intermediate risk group consists of women with a first-trimester biochemical screening risk score of between 1:300 and 1:1000. Testing for women in this group will eliminate the discrepancy between absolute and relative risk. This is important since the odds of a positive result vary with maternal age (Bhorat et al. 2018). However, first-trimester screening needs to be offered first to determine intermediate risk. As discussed previously, most of the South African population does not undergo biochemical prenatal screening beyond maternal age assessment, or previous history of an abnormal child or pregnancy. This raises concerns about the feasibility and practicality of implementing NIPT within the current South African healthcare system. First-trimester screening would have to be integrated universally into the state healthcare system before NIPT should be considered in this scenario.

#### NIPT testing is expensive and complex

When comparing the cost of NIPT with the average household income/expenditure in SA, it becomes evident that NIPT is a significantly expensive prenatal screening option. In 2024, the average price for NIPT in SA was between R5500-R6500 ($300-$330) for standard NIPT screening. According to Statistics SA, in 2022, 63% of SA households had an expenditure of less than R5000 ($270) per month (Statistics South Africa 2023a). This emphasises the cost of NIPT versus the average income in the South African population.

The SA unemployment rate in 2024 was 32% (Statistics South Africa 2024), and 49.5% of the population received government grants (Statistics South Africa 2023a). Over 9 million people in SA were medical aid beneficiaries in 2023 (CMS 2023; Statistics South Africa 2023a) with 15.7% of the South African population having medical aid. Medical aid will generally only cover the cost of NIPT when it is a high-risk pregnancy. These figures show that NIPT is only available to a minority of the South African population, as most people do not have medical aid and cannot afford it.

NIPT is a complex and resource-intensive procedure compared with other prenatal screening methods. It relies on advanced NGS technology, specialised glass collection tubes, and timely pre-sample processing. A significant limitation to the process of testing is the short viability of the cffDNA in the maternal blood sample, which must be processed within five days of blood draw to extract sufficient cffDNA for accurate testing (Diaz et al. 2016). These requirements, combined with the need for sophisticated equipment and highly trained laboratory personnel, present logistical and financial challenges, particularly in resource-constrained settings.

NIPT is usually performed at centralised laboratories because of batching requirements, expensive equipment and skills required, making it, in many cases, inaccessible in LMICs (Sium et al. 2023). NIPT is available in SA, however, samples must be sent to processing laboratories in Gauteng or abroad. Sample transportation presents a significant challenge, particularly if NIPT is to be implemented in the public healthcare sector, due to the sample's limited viability. Timely delivery to a processing laboratory is essential, yet this may be difficult to achieve in SA, where vast geographical distances and numerous small towns with limited healthcare access pose logistical obstacles. Furthermore, the transportation system itself presents constraints; for instance, it has been reported that the National Health Laboratory Service (NHLS) courier service typically collects specimens only once per day, meaning that patients still waiting to have their blood drawn at the time of collection may miss the opportunity for sampling that day (Girdwood et al. 2022).

Droplet digital PCR (ddPCR) presents a viable alternative to NGS-based NIPT, particularly in the South African context, as it is more cost-effective and offers a shorter turnaround time while maintaining comparable sensitivity and specificity (Haidong et al. 2020). Additionally, ddPCR has a simpler workflow, and both the equipment and reagents are less expensive than those required for NGS. The technology can detect subtle changes in DNA amplification, making it sufficiently sensitive for use in NIPT (El Khattabi et al. 2016).

#### Screening for other genetic disorders might be more relevant in LMICs compared with trisomy 13, 18 and 21

Some LMICs, have a higher prevalence of hemoglobinopathies including sickle cell anaemia. The prevalence of sickle cell anaemia in Nigeria is 20 per 1000 births, substantially higher than that of common trisomies. In SA the prevalence of sickle cell anaemia is less than 1% (World Health Organization 2006). One of the most common single-gene disorders that has a high prevalence in SA is Oculocutaneous albinism, which has a prevalence of 0.25 per 1000 births (Kromberg & Kerr 2022). Although the prevalence is high, it is not higher than trisomy 21. The burden of disease should guide how public health resources are allocated, with each country making decisions based on the specific health needs and conditions most common in its population.

A South African study examined the prevalence of congenital abnormalities among live births in KwaZulu-Natal Province. Among the observed abnormalities, 12.8% were chromosomal (Trisomy 13, 18 and 21). Down Syndrome (trisomy 21) had the highest prevalence of 1.73 per 1000 births, while trisomy 13 and 18 were each observed at a prevalence of 0.13 per 1000 births (Saib et al. 2021). Approximately 18% of pregnancies in SA are considered AMA (Statistics South Africa 2023b), which increases the risk for trisomy 21. Therefore, taking everything into consideration, screening for chromosome abnormalities in the SA population is reasonable and justified.

### Ethical, legal, and social issues

The ethical, legal, and social implications of NIPT are increasingly complex, particularly in a resource-constrained and socio-economically unequal setting such as SA.

NIPT should not be regarded as ‘just another blood test’. Informed consent is critical, and pregnant women must be provided with comprehensive counselling by an appropriate healthcare professional to understand the potential outcomes, implications, and limitations of the test. This includes the right to decline testing, thereby preserving patient autonomy (Smith et al. 2018). As NIPT can be performed from as early as 10 weeks’ gestation, the receipt of abnormal results may place additional emotional and ethical burdens on expectant mothers. In some cases, the availability of early information may lead to difficult decisions around termination, decisions that may not have arisen if a spontaneous miscarriage were to occur later in the pregnancy (Haidar et al. 2018). This highlights the psychological impact of early screening and the essential role of both pre- and post-test counselling.

Sex selection is a further area of legal and ethical concern. In countries where sex-selective practices are prevalent, policies have been implemented to prevent the disclosure of fetal sex following NIPT (Jayashankar et al. 2023; Ravitsky et al. 2021). In SA, however, fetal sex is disclosed if requested on NIPT screening. Although sex-selective termination is prohibited, the *Choice on Termination of Pregnancy Act* permits termination of pregnancy for any reason during the first trimester (Thaldar 2023) effectively creating a legal grey area around sex selection. Legislation regarding termination of pregnancy varies significantly between countries. Since NIPT offers early and reliable information about the fetus, it may facilitate decisions regarding termination within the legally permitted timeframe (Minear et al. 2015a).

Another legal issue surrounding NIPT includes its commercialisation (Griffin et al. 2018). The commercialisation of NIPT has given rise to marketing campaigns that often prioritise the interests of test providers over those of patients (Haidar et al. 2018). These campaigns tend to emphasise convenience, speed, and accuracy while downplaying the complexity of the test and its associated ethical and medical implications.

The social implications of NIPT access are also profound. There is potential for increased disease burden within disadvantaged populations, groups that often lack adequate resources to support individuals with special needs (Allyse et al. 2015). These disparities stem from broader systemic issues, including socio-economic inequality, institutional racism, and geographic inaccessibility, as well as barriers related to communication, cultural safety, and awareness of services (Ladak et al. 2024). As Ventura et al. cautioned, “This advanced technology should not be allowed to widen the gap between countries, or worse still between people of the same country.” (Ventura et al. 2013).

Finally, there are wider societal risks associated with NIPT, including reduced tolerance for individuals living with disabilities and increased stigma towards pregnant women based on their decisions, to terminate or not to terminate a pregnancy (Griffin et al. 2018). These issues underscore the need for policies that promote equity, ensure informed choice, and protect against discrimination, while also recognising the complex emotional and social landscape within which such decisions are made.

#### Pre- and post-test counselling and informed consent are essential for NIPT

General pre- and post-test counselling for NIPT can be provided by an appropriate healthcare professional. However, when cases become more complex, such as when a high-risk result is obtained or when greater genetic expertise is required, a trained genetic counsellor or medical geneticist should be involved to interpret the results and provide appropriate counselling. SA lacks sufficient resources for pre- and post-test counselling, with only 28 genetic counsellors nationwide (Gomes et al. 2024). The recommended target is 0.84 genetic counsellors per 100,000 population, meaning SA meets just 5% of this benchmark (Gomes et al. 2024; National Department of Health: Pretoria, South Africa, 2021). A 2017 study reported that SA had approximately one genetic counsellor per 8.4 million individuals and one medical geneticist per 4.9 million individuals (Mozersky et al. 2017). This severe shortage highlights genetic counselling as a significant limitation within the South African healthcare context. Without access to trained professionals, patients may struggle to understand, interpret, or act on their NIPT results, leaving them uncertain about the appropriate next steps.

Culture and religion play a significant role in shaping counselling practices. African proverbs, for example, often reflect the view that every child is a blessing and that children are regarded as wealth, progenitors of lineage, and an investment for the future (Musie et al. 2022). Each culture holds distinct beliefs and values concerning pregnancy, screening, invasive testing, and termination, underscoring the importance of pre- and post-test counselling, informed consent, and respect for autonomy. This is particularly pertinent in SA, a country characterised by a rich diversity of religions and cultures, where such cultural considerations must be carefully integrated into healthcare practices.

#### Unequal accessibility of NIPT

NIPT provides a reliable, non-invasive option for prenatal screening with high sensitivity and specificity; however, its accessibility remains unequal. This inequality raises important concerns regarding justice and fairness in access to reproductive healthcare.

Ventura et al. (2013) argue that “There is no reason to offer an invasive test with subsequent risk of miscarriage when there is the option of a non-invasive diagnosis with no risk. Withholding this current option from pregnant women during counselling is unethical, even for low-income families. A choice of currently available options should be offered to pregnant women regardless of the social, cultural, or economic conditions, and whether or not the test is performed in their town or country.” (Ventura et al. 2013). Although this statement was made in the context of non-invasive prenatal diagnosis (NIPD), rather than NIPT, its ethical implications remain highly relevant. It is difficult to justify offering invasive testing in the state sector, which carries a risk to the pregnancy, when a safer alternative exists, particularly if the only barrier to access is financial.

In SA, NIPT is currently accessible only to pregnant women who pay out-of-pocket or those with high-risk pregnancies covered by private medical aid. Therefore, pregnant women in the public sector cannot access this test unless they pay out of pocket. In the public sector, a pregnancy that is deemed high risk due to AMA, blood tests, previously affected pregnancy or ultrasound assessment will receive an invasive test if required and provided that resources are available (Bhorat et al. 2018).

Accessibility is also dependent on location. Since NIPT technology is expensive, the test is centralised at specific laboratories. Therefore, NIPT might not be offered in small towns in SA, as they will not have the correct collection tube and/or transport necessary to ensure the sample reaches the laboratory promptly.

The South African Society of Obstetricians and Gynaecologists (SASOG) and the Gynaecology Management Group (GMG) stated the following:

- “If money wasn’t an issue and there were more than enough experts in the country, the very best screening would be a combination of NIPT with an expert Nuchal Translucency (NT) scan and an expert fetal anatomy scan and a repeat expert scan in the last trimester. This combination could theoretically detect 99% of all Down syndrome fetuses, a whole list of other genetic conditions, as well as the majority of physical fetal abnormalities. This approach, even though some patients may choose this, is very expensive and will not be available for all pregnancies soon. This is not only the situation in SA but also in many developed countries. For this reason, alternative screening strategies can be considered acceptable, with some form of triaging according to risk.”(The South African Society of Obstetricians and Gynaecologists & Gynaecology Management Group 2018)

## Limitations of study

This scoping review was limited to articles published in the English language. As a result, relevant studies published in other languages**,** particularly from LMICs where English is not the primary language**,** may have been inadvertently excluded. Additionally, the process of thematic analysis was guided by the authors’ interpretation of what was considered relevant and important to the review objectives. Consequently, it is possible that certain themes or issues were overlooked or underrepresented. Given the limited volume of available literature, the impact factors of individual journals were not considered in the selection process.

## Conclusion

The current landscape of NIPT in SA is both complex and underrepresented in the scientific literature. Existing South African publications are limited and largely outdated, with few recent contributions of significance.

International guidelines, such as those issued by the American College of Obstetricians and Gynaecologists (ACOG), recommend that all pregnant women be offered prenatal screening before 20 weeks’ gestation, with the option of invasive testing irrespective of maternal age. However, in SA, as in many other LMICs, such recommendations are difficult to implement. Access to prenatal screening is often dictated by the availability of resources, resulting in unequal access to more advanced screening technologies such as NIPT (Bhorat et al. 2018). In a setting where financial constraints dictate healthcare choices, the more advanced screening methods often remain out of reach for many expectant mothers.

While NIPT is globally recognised for its accuracy and non-invasiveness, its reliance on NGS technology, associated high costs, and technical demands render it largely inaccessible within South Africa’s public health context.

Implementing NIPT within the public healthcare sector remains highly challenging, particularly given that even basic prenatal screening is not routinely offered due to systemic resource limitations. SA requires a screening test with all the benefits of NGS NIPT but without the cost. There is a need to develop, verify, and implement a cheaper NIPT option for people paying out of pocket and for the public sector (Minear et al. 2015b). Droplet digital PCR-based NIPT (ddNIPT) presents a promising alternative, which offers comparable sensitivity and specificity to NGS-based methods but at a lower cost and with a simplified workflow (El Khattabi et al. 2016).

Despite the well-known limitations of NIPT in SA, the benefits of the test outweigh these drawbacks. Given the challenges in the public healthcare sector, the authors propose offering ddNIPT to high-risk patients early in pregnancy as part of a pilot study recommended by the Clinical Guidelines for Genetics Services in SA (National Department of Health: Pretoria, South Africa 2021). Unlike costly and complex NGS-based NIPT, ddNIPT is simpler and more affordable, making it a more feasible alternative for managing high-risk pregnancies in a low- to middle-income setting. This approach could potentially reduce the need for invasive diagnostic procedures and improve prenatal screening, making NIPT more accessible and achievable in SA.

Collaboration between and within the public and private healthcare sectors is essential to improve access to prenatal screening in SA. Strategic partnerships should aim to identify and evaluate cost-effective alternatives to NGS-based NIPT, as well as to enhance existing prenatal screening methods. Such collaboration would not only facilitate the development and implementation of more accessible technologies but also foster local research and knowledge-sharing between sectors.

## Future research and recommendations

Future research should focus on the clinical validation of ddNIPT in the South African setting to determine its viability as an alternative to NGS-based methods. Additionally, there is a clear need for more locally generated evidence, including peer-reviewed publications, case studies, and implementation research that reflect the country’s specific clinical and socio-economic context. Building a robust body of South African NIPT research will be key to informing policy development and ensuring context-appropriate, equitable prenatal care.

## Supplementary Information

Below is the link to the electronic supplementary material.

Supplementary file1 (XLSX 45 KB)

## Data Availability

No datasets were generated or analysed during the current study.

## Abbreviations

*ACOG*: American College of Obstetricians and Gynecologists
*AMA*: Advanced maternal age
*cffDNA*: Cell-free fetal DNA
*CVS*: Chorionic Villus Sampling
*ddNIPT*: Droplet digital PCR NIPT
*DNA*: Deoxyribonucleic acid
*GMG*: Gynaecology Management Group
*HICs*: High-income countries
*HIV*: Human Immunodeficiency Virus
*LMIC*: Low- or middle-income countries
*MTCT*: Mother-to-child transmission
*NGS*: Next-generation sequencing
*NIPD*: Non-invasive Prenatal Diagnosis
*NIPT*: Non-invasive prenatal testing
*NT*: Nuchal Translucency
*PCR*: Polymerase chain reaction
*SA*: South Africa
*SASOG*: The South African Society of Obstetricians and Gynaecologists
*SASUOG*: The South African Society for Ultrasound in Obstetricians and Gynaecologists
*TOP*: Termination of Pregnancy
